# School Surgery

**Published:** 1895-12

**Authors:** Arthur W. Prichard

**Affiliations:** Surgeon to the Bristol Royal Infirmary, and Lecturer on Practical Surgery, University College, Bristol


THE BRISTOL

### Medico-Chirurgical Journal.

DECEMBER, 1895.

### SCHOOL SURGERY.

The Presidential Address, delivered on October 9th, 1895, at the opening of the 22nd Session of the Bristol Medico-Chirurgical Society,

BY

Arthur W. Prichard, M.R.C.S. Eng.,

*Surgeon to the Bristol Royal Infirmary, and Lecturer on Practical Surgery, University College, Bristol.*

In taking my place as your President, my feelings are naturally of a mixed character. On the one hand, I, in common with other members of my race, duly appreciate an honour, and I sincerely thank you for this one—one of the greatest local medical honours that the profession can bestow upon one of its members; and on the other hand, there is the fear that I may not be able to do adequate justice to the position in which you have placed me. I crave your indulgence if in my address, or in any of the duties that fall to the lot of the President, I come far below the mark of excellence that heretofore has generally stamped the occupier of this Chair. For my own part, I promise to do my best; and judging from the vigour displayed by the Society of late years, I think I may confidently hope that during my year of office there will be no falling off, either in the number of attendances at the meetings or in the high standard of the papers and discussions over which it will be my pleasant duty to preside.

In July, 1891, Mr. Glazebrook appointed me surgeon to Clifton College; but for twelve years before then I had done some of the surgical work for my father, who had held the post since the College began. I therefore consider that I have been connected with the school long enough to enable me to make a paper on "School Surgery" worth your hearing, and for me to speak not without some authority of the accidents and other surgical emergencies that have occurred in one of our largest public schools during the last few years. The subject is one that, even if it does not involve any great scientific research, may prove itself to be of some practical utility, and, considering the pleasure that the great majority of our profession take in athletics, may not be altogether without interest.

Of rowing I have nothing to say, as there is no river here accessible to the boys, and I shall confine my remarks almost exclusively to my personal experiences of football, of runs and athletics, and lastly of cricket. I must make a fourth section of curious accidents and some operations and other conditions that have come under my notice, originating in none of these sports.

Beginning with the present term, I shall mention some of the accidents that happen from football; and at the outset I cannot but say that to one watching a game of football under Rugby rules, on the result of which depends some great stake such as the honour or position of the school or house, it is marvellous that serious mishaps do not occur more frequently than they do. Fifteen boys, chosen for their weight, strength, quickness, or cleverness, are bent upon gaining an advantage over fifteen similarly-picked individuals, and for an hour and a quarter the members of each side play all they know and strain every sinew to secure the victory. Of course a certain amount of good-natured violence is exhibited, and many are the collisions, falls, wrenchings, collarings, and throwings that happen, and yet most games finish with no one complaining of very serious hurt.

At school, football is somewhat different as regards its surgical results from football played by full-grown men—at least, such is my experience. At school, fractures are comparatively rare: the most common injuries are to the muscles, and of all the body the muscles of the thigh suffer most. "Sprinter's sprain," or sprain of the flexor muscles just below their origin from the tuberosity of the ischium, is not uncommon, and very troublesome, compelling the patients to lie in bed or to walk quite stiffly for two or three weeks; but I think that giving way of some fibres of the quadriceps extensor, with its characteristic signs, is more common. This may be simply from a hard muscular effort, but I have no doubt that it more frequently occurs by the thigh coming into violent contact with an adversary's head or knee, or other part of his body, while the muscle is contracted in running. The effect is loss of power of the muscle with pain in one spot and immediate swelling above it, giving evidence of rupture of some of its fibres. This condition is sometimes slight and the boy makes no complaint; but a few days later, when attempting to run, more of the damaged muscle gives way, and the result is that the player is incapacitated. The frequency of this accident is remarkable, and it occurs not uncommonly more than once in the same individual, showing that there must be some natural weakness of the muscular fibres. I think that these cases of rupture of muscular fibres are more common at the beginning of a season than later on, when the muscles from constant and regular exercise are in a harder and better condition.

Allied to this accident is one of separation of the anterior inferior spine of the ilium by muscular action, of which I have seen three instances. Very little notice has been taken of the condition in systematic works on surgery, but it is one that presents most definite signs. The anterior inferior spinous process is an epiphysis that appears about puberty and is not completely united with the rest of the bone until the twenty-fifth year; it is the origin of the straight tendon of the rectus femoris, and it is doubtless the forcible contraction of the rectus that causes the accident. The cases were all characterised by the same symptoms, which were loss of power in the thigh and a sensation of something having given way near the hip during a violent muscular effort. I may take for instance the details of the most recent case. A boy, aged fifteen and a half, thin and overgrown, on the third day after coming back from the holidays, while playing punt-about, took a drop-kick at the ball and missed it. Whether the abortiveness of the kick was the cause or effect of the accident, I cannot say—the latter, I think. He was able to walk to his house, and I was not called to see him till next day. Then there were pain and swelling in front of the hip-joint, and a hard mass could be felt in the tendon; and on flexing the limb on the body, soft crepitus could easily be felt by moving the fragment sideways. The other cases had the same distinctive signs. Rest in bed in the flexed position and bandaging was the treatment used, and the patients were about again in from three to five weeks. There was no evidence of any permanent weakness.

I have seen a case presenting very similar symptoms in a boy aged seventeen. He felt something give way near his hip, and there were thickening and shortening of the fleshy part of the rectus, with inability to raise the leg from the bed. In him I believe one tendon of the rectus was lacerated near its origin, and in this case the pain and weakness lasted a longer time than in the cases of fracture previously mentioned.

Of other muscles or tendons giving way or being badly stretched I have seen many instances—notably, a rupture of the flexor tendons of the wrist from a fall on the hand; two cases of stretching of the flexor tendon of the ring-finger by its being forced backwards; a distinct case of rupture of the gastrocnemius, probably caused by a hack from behind; and many cases of strain of the erector spinæ, sometimes with and sometimes without swelling of the muscle of one side. This in football is possibly done by the violence of being caught round the waist and thrown. One case, however, occurred in a boy who was playing Association football in the spring, and who came under my care when he came back for the summer term. In this term he quite recovered and played cricket, but the first game of Rugby football in October brought back all the old symptoms of injury to the erector spinæ and he was incapacitated.

Bruises and blood-tumours under muscles I will pass over, though some of the latter give rise to much pain and inconvenience—especially those under the pectoralis major and the vasti as they cover the condyles. Effusion of blood among the muscles of the thumb in one case gave a great deal of trouble. Hæmatoma of the ear is a very common affection caused by football, occurring chiefly among the forwards, whose ears get a good deal crushed in the scrimmages. It is an unsightly condition, and one that, if not successfully treated—and success in its treatment cannot be always ensured,—leaves the patient with one very ugly ear for the rest of his life. The treatment which I have found most generally useful is to let out the contents of the tumour by one or more incisions—the contents being often clear serum—and then to prevent its re-forming by keeping a wooden clip on the pinna, which exerts gentle pressure for a day or two. If the contents suppurate, as they sometimes do, of course the pus must be evacuated and a drainage-tube kept in; but I believe that this nearly always results in an unsightly thickening.

Of dislocation of the larger joints from football I have had no experience: dislocation of the patella occurs, but is generally reduced by the player himself or his friends immediately on receipt of the injury. I have seen some cases of dislocation of the thumb,—two in one day, curiously,—and many cases of dislocation of the joints of the fingers, and they have occurred in picking up the ball in a charge; and I may say here that a great many more of the smaller accidents occur in a loose scrimmage than when the players are packed together. I have seen two or three cases of partial dislocation of the outer end of the clavicle, a condition from which, in my opinion, perfect recovery seldom takes place.

Fractures are decidedly rare. There was one case of a broken leg at the College last year, and I can recall several others, but not very recently. Fracture of the clavicle is the commonest, and next to that fracture of the forearm; among the smaller boys this is, as one would expect, often of the greenstick variety. I have heard the snap of a fractured ulna when I have been standing close to a game. A broken nose sometimes comes under observation, and a broken rib is not unknown; and I have seen a case of fracture of the lower end of the sternum from Association football, but not at the College.

One alarming accident was a rupture or fracture of the trachea. When I saw the boy there was a tender spot about three rings below the cricoid, but no bruising; the throat, neck, face, and chest were quickly emphysematous, and there was great difficulty in swallowing but not in breathing. Fortunately these symptoms began to improve very quickly, and in ten days had entirely disappeared.

Sprains of the ankle are very common, as one would expect, but sprains and injuries of the knee-joint are nearly as common and much more serious. Effusion into the knee after a sprain and giving rise to considerable trouble occurs more commonly than it would if boys could be made to look upon a sprain of the knee as a serious injury that requires rest from the first. I have had some cases of the joint becoming full of blood, causing a great deal of pain, and I have had cases of persistent tenderness along the edge of the semilunar cartilage; but the common result of knee-joint injuries is, to find the patella floating and the *cul-de-sac* above the knee distended with fluid. In these cases I may say, and I know it is at the risk of saying nothing new, that there is no plan of treatment half so efficacious as to blister the knee all over while it is kept at rest on a McIntyre splint, and to make the patient wear an elastic bandage or knee-cap directly he is able to do so.

As regards flesh-wounds, there is no special feature in them arising from football; the custom of hacking has gone out, and now one does not often see a serious break of the skin below the knee. The time was when it was the exception for a football player to have an unwounded shin, and many can show the scars of wounds caused long ago. The ankles sometimes get cut, but chiefly by players on the same side as the hurt player, the wound being commonly behind the malleolus. Scalp wounds are not uncommon.

I cannot pass over a case of periostitis of the tibia with suppuration that I had in the person of one of the pluckiest players in the school. He had had repeated kicks in several matches on the same part of the front of the tibia, and when he applied to me on account of pain in his leg there was an abscess under the periosteum, about four inches above the ankle. This was opened and drained, and although sinuses formed, and took some time to heal, yet complete recovery took place without necrosis, which would probably have occurred had the incident happened to one in the lower ranks of life.

I had hoped to have drawn some definite comparison between Rugby and Association football from a surgical point of view, but my experience of the latter hardly justifies it. I am inclined to the opinion that, as regards injuries to muscles and joints, Rugby is the more dangerous, while Association accidents are fewer, but when they occur are often serious. Cuts from collisions, and fractured and otherwise injured legs from cross-shins, are the most common. I will mention two curious accidents that I have seen as the result of Association football. One was a severe kick in the face that a man received who was trying to head the ball when his adversary was making a high kick at it, and the other was a cornea cut across by the ball. This, I believe, must have been done by a loose tag. The result was loss of the eye.

In the spring term, from January to April, the long runs take the place of football, and towards the end of this term the athletic sports come on; and I propose now to deal with certain accidents that occur in the runs and in the practising for the athletics. On the subject of runs there is from a medical point of view a good deal to be said, but not officially by me. The medical examination of each boy, the question of his strength and of the healthiness of his heart and lungs, belong to the physician of the school, under whose care also are placed the cases of exhaustion amounting almost to collapse that occasionally occur as the result of a race such as the Long Penpole run. Without trespassing on the province of my friend and colleague, Dr. Fyffe, I may give my personal opinion as to the value of the runs, which is that they are of more good than harm, and in healthy boys very seldom cause any mischief. The value of the lesson of endurance that the runs teach more than compensates for the fatigue and the few accidents they cause. Were they abolished or much diminished, as many parents wish, the general result would probably be an increase in the amount of ill-health. It is a cold and damp season of the year, and the runs keep the boys in training better than other games would. Other games provided for so large a number would probably involve standing about by some boys, and this would be dangerous; and if no systematic active exercise were enforced, the demoralising effect of loafing would be sure to make itself evident in the present satisfactory tone of the school.

Surgically from runs I have had to deal chiefly with the legs and feet. Corns are peculiar and very troublesome; they take the form of large flat thickenings of the epidermis on the tread of the foot. They are not as a rule taken serious notice of till they become inflamed, and then they sometimes suppurate. The frequency of the occurrence of these flat corns makes me think that they are caused by the India-rubber sole of the running-shoe being too flexible, and so the weight of the body is concentrated on the part of the sole just behind the web of the toes, instead of being spread over a larger area as it is when leather soles are worn.

Frequently flesh-wounds on the leg occur from jumping into a hedge or ditch where there is a hidden stake or broken stump, and these wounds present a curious similarity. They consist of a V-shaped tear with the apex downwards. There is always a great retraction of the flap and generally some foreign material is present in the wound when it comes under observation, so that it is no wonder that they do not heal very readily. One peculiar case was as follows. A boy thought he hurt his thigh in getting through a hedge, but ran home and did not complain till next day, when I removed a small piece of wood from a very small wound in the front of the thigh. As there was afterwards more thickening than could be accounted for by cellulitis arising from the small foreign body, I gave him chloroform and made an incision, and drew out a piece of blackthorn quite three inches long.

Sprains are of course very common, chiefly those of the ankle-joint; but I have had many cases of sprain of the transverse joint of the foot to which I should like to draw attention. The condition, started by landing from a jump on uneven ground in thin shoes, is made much worse by the boy continuing to run, or perhaps going for another a day or two after. Pain and slight swelling in the neighbourhood of the astragalo-scaphoid joint appear, and however carefully the case is treated it is a long time before complete recovery takes place. Indeed, I may say in this place that as a rule sprains are generally very much underrated as to their consequences, and if any liberty is taken with the injured joint before it is quite well, a relapse of the symptoms is likely to take place which incapacitates the individual from active exercise for a considerable time. It is doubtful indeed whether a simple uncomplicated fracture of a long bone is not a less serious injury than a bad sprain.

Teno-synovitis of the peronei and other tendons sometimes results from a run, and I have had a troublesome case of sprain of the external lateral ligaments of the knee, a fractured fibula, two or three cases of strain of the abdominal parietes and diaphragm, and many cases of abrasions and blisters. These form the great majority of the casualties that have occurred, and I have had to stop boys from running on account of flat-foot, varicose veins, and hammer-toe.

I shall now record one or two results of the athletic sports, or of the practising for them, which have come under my notice. Sprain of the muscles of the back of the leg or thigh in starting to run or jump is common, but I have already alluded to that in speaking of football. One troublesome case from jumping was a strain of the external rotator muscles of the hip. Jar of the heels in the long jump is a painful condition for a few days, but not serious; and falls over the hurdles occasionally cause damage.

I must mention more in detail a case of fracture of the patella, which is interesting on account of its cause. A boy just six feet high and a few days under fifteen years old, and a favourite for the high jump under fifteen, ran to take off for it. He was seen to rise very little from the ground, and to fall "in a heap." Mr. W. G. Grace, who was close by, rendered first aid, and the boy was carefully carried to the Sanatorium. We found a transverse fracture of the patella, the upper fragment of which was pulled up three inches, and the lower or smaller fragment comminuted by a longitudinal split. After several consultations, and explanations of the nature of the operation of wiring the fragments and of the statistics of the results of recorded cases, the parents, contrary to my advice, decided against any cutting operation. I therefore treated the case by splints and apparatus. By continuous weight-extension on the muscles of the thigh, after three weeks there was a gap of three-quarters of an inch, and I never could improve upon that. He was allowed up five weeks after the accident, and the pressure was kept up by lacing over a gap in a leather splint. At the present time, eighteen months after the accident and six months since all splints have been left off, there is an interval of 1¼ inches between the fragments; he is able to walk with almost a natural gait, swim easily, and can trot but not run fast. In this case I believe wiring the fragments would have greatly shortened the treatment and have given a more satisfactory result.

Another case of fracture is worth recording, even if it is only to excite sympathy for the unfortunate individual to whom it occurred. The next jump to the one I have referred to last was taken by a boy aged 14½, and he fell and broke his radius. The high jump competition was therefore stopped for the day. This occurred at the end of the spring term, and the broken arm spoilt the boy's Easter holidays. However, he was well enough to play cricket during the last half of the summer term. The first day of the summer holidays he fell off his bicycle and broke the same arm again. He recovered from that; but at Christmas, in doing a banister slide, he fell and broke the same arm again higher up, and he thus spoilt nearly the whole of the pleasure of the year's three vacations by three times breaking his arm.

We come now to the summer term and the accidents and injuries that may happen from cricket, a game that is remarkably free from danger. Considering the huge number of people who now play the game, and the sometimes great pace of the bowling, and that too on a wicket that is the reverse of perfect, I think it is curious that so few cases of serious injury occur. At a school with a carefully-kept playground, it is not generally in a match that we see accidents. They occur most commonly during practice at the nets, and when many games have to be played in a limited area, and therefore the fieldsmen of one game are near, or perhaps overlapping, the fieldsmen of another. An unexpected blow from the ball frequently occurs, though not commonly resulting in much harm. I have had, however, under my care from this cause many contusions, two or three cases of concussion of the brain, and at least one case of fracture of the skull. In formal cricket the commonest accident that might be called serious is, I think, dislocation of the top joint of the fingers in catching or stopping a hard hit. This results in perfect recovery after a few days inconvenience, provided the dislocation is reduced at once; but I have seen a case in which permanent stiffness of the joint ensued because the boy let the joint remain out for a fortnight before he sought surgical assistance. In a ladies' match last year one of the players at a school in Clifton attempted a catch at short-leg, and the result was a bad compound dislocation of the middle finger and a simple dislocation of the ring-finger. This hit was by a girl considerably her junior. Next in frequency perhaps is the more or less split web of the fingers, and I believe it always arises from the clumsy way in which a fieldsman attempts a catch. If his fingers are up towards the ball,—and the accident happens more often from a lofty hit than a low one,—the ball may come down between separated fingers and so split the web. The ball should be received into the palms of the hands. Injured knuckles and fingers from playing fast bowling without gloves, and blows on the thighs above the pads, injuries from collisions, broken noses and cut lips, are common and need no special comment.

The most important surgical question that arises from cricket is, how far a growing boy ought to tax the strength of his muscles and frame in bowling. Bowling with a high-reaching delivery and as fast as possible is a frequent source of strain of the muscles of the back. If the bowler is right-handed, the trouble appears in the left erector spinæ and quadratus; and when once it has appeared, it is sure to recur if a considerable rest from that kind of bowling be not enforced. It is easy to see that lateral curvature may be induced, and I have seen cases where, if it has not been begun, it has been made materially worse by this practice. Some instances of sprain of the scapular muscles have come under my notice also, from bowling and from throwing. The question of bowling by young boys is one that ought not to be lightly passed over. The ball is too heavy and the pitch is too long, in my opinion, for most boys below the age of thirteen or fourteen, and boys can be taught to play with more precision if a smaller ball is used and a shorter pitch.[1] It is painful to see a small boy with the ordinary cricket ball and at the regulation pitch go on, over after over, trying to bowl as fast as his strength will let him, and I am sure it would be far better for the boys' own sakes, whether as regards their general well-being or their future cricket career, if some modification were made in the laws of cricket, defining a certain weight and size for the ball and a certain length of pitch for matches between boys under the age of fourteen.

In preparing the cricket part of my paper, although I do not wish it to be understood that he is in any way responsible for the opinion I have just expressed, I have asked to give me his experiences of accidents in the cricket field the greatest of living cricketers, who, among the many records that he has broken, holds the world's record—at all events, among medical men—of having his name known to the largest number of English-speaking people in the universe. He with us all rejoices at the immunity from serious injuries which cricket enjoys. One injury, he says, that occasionally occurs, and one which he has suffered from himself, is, that in starting from the crease to make a sharp run a few fibres give way in the gastrocnemius, making the player feel as if he had been hard hit on the back of the leg. This quite incapacitates the player for the time from running, and the best treatment, although

[1] This I am pleased to hear has been the custom in the Junior School at the College for some time.

very painful, is to have the part strapped with firm plaster, or to have an elastic bandage put on. Indeed, for many cricket injuries an elastic bandage is a very useful item in one's cricket bag. Mr. W. G. Grace has seen one death in the field, and that was a sad case of a boy at Harrow who received a blow on the head, while umpiring, from a ball hit to leg by a batsman of his own game, while he was paying too much attention to a more important game played close by. Another death, but not on the cricket field, occurred a few years ago in the Notts *v.* M.C.C. match. A professional player was stunned, but insisted next day in travelling home, saying he was not seriously hurt. Coma came on and he died before he reached home, presumably from hemorrhage. On August 22nd this year, a player at Clapham when batting was struck in the neighbourhood of the heart and died in a few minutes.

A curious case that Mr. Grace mentioned was that of a Kent gentleman, who, in playing at Gloucester against Gloucestershire, jumped at a catch, while fielding at point, and dislocated his shoulder. The following year, in the same match but at the County Ground, Bristol, he fell while fielding and met with the same result. Mr. E. M. Grace brought him at once to the Infirmary, where I happened to be on duty, and I reduced the dislocation. Notwithstanding that he had to be put fully under chloroform to effect the reduction, the Kent player insisted upon going back to see the match an hour after he had come round. One case Mr. W. G. Grace cited as showing the pluck of some players was this. A wicket-keeper dislocated the top joint of his finger so badly that the skin was split across. This was reduced and a bandage put on. He then resumed his place at the wicket, and did his work with credit till the end of the match. Bad blood-blisters are common, and sometimes he has seen broken metacarpal bones, but never any serious damage to the abdomen or to the scrotum. This last fact rather surprised me, as a blow upon this part of the body of the batsman or wicket-keeper is apparently so frequent.

Leaving cricket and coming to the fourth and last section of my paper, I wish to detail one or two serious accidents and to make a few remarks on other occurrences that have taken place at the College, and that have not originated in any of the games I have previously mentioned. One accident which at one time looked as if it might have a very serious termination occurred in this way. The boys in a certain house, under the supervision of the house-master, were having practice in the use of the fire-escape, and were using that kind of escape which consists of a long canvas bag, down which the boys shoot one after the other. They ought to swing from the bar by which the apparatus is attached to the window, into the canvas passage, but the boy of whom I am writing let go his hold too soon and struck the lower part of his spine violently against the window-sill. He went down the bag safely and could walk afterwards, and although in pain, did not complain to the house-matron till next day. Great pain in his back and abdomen then suddenly came on, and there was copious bleeding from the rectum and some spitting of blood. When I saw him soon afterwards the abdomen was swollen and tender, with dulness on percussion just above the crest of the ilium on the left side and in the left loin—some blood was coming from the rectum, and on examination *per anum* I could feel a considerable effusion of blood. By rest in bed, with the help of opium, this all disappeared, and in ten days time he was comparatively well. The boy was in an excellent condition of training, and I attribute his speedy recovery from these dangerous symptoms partly to this cause. Another accident worth mentioning was that of a boy who was trying to circle—as if it were a horizontal bar—one of the nets that are stretched between the trees to prevent the ball going over into the road. The net gave way, and he fell a few feet on to his back on the grass. He was perfectly well and strong before. After the accident there was pain in the region of the 10th and 11th dorsal spines, but below the 10th there was a gap and the 11th was nowhere to be felt. He complained of slight numbness and tingling of one leg, but there was no paralysis. I applied a plaster-of-Paris jacket. After three weeks a London specialist was called down to see him. This gentleman was of opinion that the case was one of congenital absence of that particular spinous process where the boy was hurt. It is a curious coincidence that the fall led to the discovery of such a rarity. It is the only case of absence of one of the dorsal vertebral spines that I have ever seen.

From the gymnasium I have had a few accidents, but none to call for special mention of their details. There was a bad fracture of the surgical neck of the humerus from a fall, and a very painful strain of the muscles and fasciæ in front of the chest from the boy going down between the parallel bars. I have also seen a case of considerable effusion in the elbow-joint brought on by boxing. One accident in the swimming bath must be mentioned. A boy slipped off the edge of the bath, and somehow his foot caught inside a handrail that runs round the bath just above the water; his ankle was fixed between the rail and the wall, and his body fell into the bath. Instead of the leg breaking, the soft parts were so crushed that a few days later large bullæ formed, and the whole aspect of the limb was as if gangrene had set in. However, after two or three weeks anxious nursing, the inflammation went down, and the boy recovered by the end of the term; but he broke his leg riding a pony in the ensuing holidays. In the chemical laboratory cuts with broken glass, and splashes of acids and other fluids in the eye, occur, but I have not had any case of real importance.

From fives, bat-fives, and racquets I have not had many patients. Sprains of the hips and injuries to the finger- and thumb-joints from hard hitting, and a peculiar sprain of the shoulder, are most common in hand-fives. This sprain of the shoulder is so often complained of that it ought almost to be dignified by the name of the fives shoulder, in imitation of the lawn-tennis elbow. It consists of a sprain of the supra- and infra-spinatus muscles and triceps, and gives a great deal of pain down the arm. From racquets I have seen two serious cases—one of concussion from a blow by the ball on the temple, and another a bruise of the globe of the eye and lid which gave rise to considerable trouble. The ball was hit from behind and glanced off the handle of the front player's racquet and hit his eye. There was ecchymosis of the lid and sclerotic, intense photophobia and dilatation of the pupil; there was no intra-ocular hemorrhage. The dilatation was still present when the boy left four weeks later. He had not been able to use his eye at all without pain.

Though golf hardly comes under the category of public school games, I may just mention it in order to point out that there are two common injuries incidental to it. One of these is a troublesome cramp of the forearm from holding the club too tightly, and which when it comes on at a critical moment often spoils the stroke; and the other is effusion in the elbow-joint from the jar of hitting the ground in bad hits. This is probably only experienced by a novice or a clumsy player of the game. I have also seen cases of strain of the left side of the back from hitting.

The tendency in most of the boys is to make the best of any injury, so that they may be kept out of school and therefore out of games no longer than is absolutely necessary. But of malingering to shirk work at school I have had a few amusing instances. One small boy had an abrasion of his shin which resisted all our attempts to get it to heal, and it was found that he kept the wound open by brushing it with some developing solution for photographs. In another term the same boy frightened us all by telling the matron that the bulb of the thermometer which she had placed in his mouth had broken off and that he had swallowed it. Knowing his previous history, I ordered a four days quarantine with very close observation; at the end of which time, as neither the bulb nor any symptom appeared, he was sent back to school properly punished.

In referring to surgical maladies other than accidents or their consequences, I shall merely enumerate the cases that have come under my care, because they present nothing that especially needs comment. Where there is any chronic disease or condition that requires surgical aid, the chances are that the boy is kept at home for treatment, and I have had therefore at school to deal chiefly with emergencies. Hernia is not very uncommon, but I have had one case of herniotomy for strangulated hernia that I did for my father some years ago. This boy was in the eleven next year. I have seen instances of orchitis during the development of the organ, and of mastitis from the rubbing of the brace-buckle. Retention of urine from nervousness I have seen, but I have never passed a catheter, the sight of the instrument being enough to bring about the desired result. I have several times had to remove the tonsils and to circumcise. One case of tracheotomy, one case of aspiration for pleurisy, two cases of draining the chest for empyema, one case of removal of a piece of glass that had been embedded in the hand for twelve months—these, besides opening abscesses and other trifling operations, form the list of all the operations that I remember to have done on the College boys. Ingrowing toe-nail is one of the smaller troubles with which one not infrequently meets. A great many ear and eye diseases come under my notice, and I know of no more anxious case than a bad one of suppurative disease of the middle ear: we had an epidemic of it at the College three years ago. Of the eye diseases I may have something to say at a future time. I have not seen an outbreak of ophthalmia, but there was one before I was officially connected with the school. The number of boys whose refraction is abnormal is very great, and one has to be careful that the vision of each boy that is going to be prepared for examination for entrance into any of the Services is good enough to pass the Medical Board before he goes on with the special education for that service. Testing for spectacles, giving anæsthetics for the dental surgeon, and revaccination form no small portion of my work.

In conclusion, I feel that my remarks would be incomplete if I did not offer an opinion as to whether the advantage gained by the boys from the games I have mentioned is worth the risk of accident that they run. I say that decidedly it is worth the risk. I have necessarily brought into prominence results of many accidents, but I do not wish it to be understood that accidents are unduly common. The great majority of the boys at the College meet with no serious hurt, and have never come under my care at all; and when we think of the immense gain to the health and strength of body, mind, and character of the boys that the games give, the chance of accident sinks into comparative insignificance. There are no outdoor games that are perfectly safe, and the very fact of there being an element of danger, especially in such a game as football, not only lends the excitement that makes the game worth playing, but also engenders the qualities of pluck and self-sacrifice that make some English boys what they are. If asked whether any change should be introduced, beyond the hint that I have given above as to the care that should be exercised over the younger boys in learning to play cricket, I would say that no change is necessary. In football I think it important that boys of about the same age should play together, and I believe that that plan is carried out in all large schools. Personally, I think, and I believe the great majority of the profession will agree with me, that the school authorities are right, not only in encouraging, but in enforcing among able-bodied boys the games that are at present the custom.

I thank you for listening to this address, which I am afraid has been long, and possibly I may have sorely taxed your patience by going too much into trivial details. I have endeavoured only to call attention to the surgical incidents that fall to the lot of a school surgeon, and I have avoided almost all reference to treatment. I must say that, thanks to the good general health of the boys, the excellent hygienic condition of the College, and the careful nursing of the matron at the Sanatorium, the results of the treatment are most satisfactory, and would bear a favourable comparison with those met with in hospital patients of a different class of life.

